# A simple and efficient method for cytoplasmic production of human enterokinase light chain in *E. coli*

**DOI:** 10.1186/s13568-022-01504-9

**Published:** 2022-12-27

**Authors:** Mohammad Ebrahimifard, Mohammad Mahdi Forghanifard, Ahad Yamchi, Vajiheh Zarrinpour, Mahrokh Sharbatkhari

**Affiliations:** 1 Department of Biology, Damghan Branch, Islamic Azad University, Damghan, Iran; 2 Department of Biotechnology, Gorgan University of Agricultural Sciences and Natural Resources, Gorgan, Iran; 3 Research and Development Division of Arya Tina Gene Company, Gorgan, Iran

**Keywords:** Recombinant enterokinase, *Escherichia coli*, Response surface methodology, Protein folding, Affinity chromatography

## Abstract

Human enterokinase light chain (hEKL) cDNA sequence was designed with the help of codon optimization towards *Escherichia coli* codon preference and ribosome binding site design and artificially synthesized with a thioredoxin fusion tag at the N-terminal and a five his-tag peptide at the C-terminal. The synthetic hEKL gene was cloned into the pET-15 expression vector and transferred into the three different expression strains of *E. coli* BL21(DE3), NiCo21, and SHuffle T7 Express. Different growth and induction conditions were studied using a statistical response surface methodology (RSM). Recombinant hEKL protein was expressed at high levels in soluble form with 0.71 mM IPTG after 4 h of induction at 25 °C. Autocatalytic process cleaved TRX tag with enterokinase recognition site by the impure hEKL and yielded the mature enzyme. The target protein was then purified to homogeneity (> 95%) by affinity chromatography. The activity of hEKL was comparable to the commercial enzyme. From 1 L culture, 80 mg pure active hEKL was obtained with the specific activity of 6.25 × 10^2^ U/mg. Three main parameters that help us to produce the enzyme in the folded and active form are the type of strain, SHuffle T7 strain, TRX and histidine fusion tags, and growth conditions including the increase of OD of induction and IPTG concentration and the decrease of induction temperature.

## Key points

1. Combining several parameters suggested in different papers in one experiment to get the active enzyme.
2. Decreasing the temperature of induction and the amount of inducer and using a chaperone-like tag simultaneously to help the protein folding system of the bacteria.
3. Using a statistical method (RSM) to analyze a number of treatments and prediction of the most effective ones to optimize the expression system.

## Introduction

Fusion expression can often lead to an efficient expression of the target protein. A lot of recombinant protein has been produced using different fusion tags. The expression of recombinant enzymes without fusion tags in *E. coli* is either prone to degradation or misfolded form (Ki 2020). The fusion tag is removed from the target protein by protease with the specific recognition site between the target protein and the fusion tag. Enterokinase is regarded as an ideal enzyme to prepare target protein by sequence-specific cleavage from the fusion protein (Liew 2005).

*E. coli* with a simple, rapid, and economic growth rate and efficient scalable process is the first choice for an expression system. However, most heterologous complex proteins with multiple disulfide bonds expressed in *E. coli* are prone to be insoluble and inactive in the form of inclusion body (Chun et al. 2011; Hayat et al. 2018; Kaur et al. 2018), which often leads to inefficient in vitro refolding techniques to obtain biologically active conformations. The use of special fusion tags by linking the gene of interest to the tag sequence which is already known to be expressed soluble well in *E. coli* is a popular strategy to maintain the expressed protein in the soluble form (Liew et al. 2005).

The light chain of the enterokinase as the catalytic subunit consists of a chymotrypsin-like serine protease domain that recognizes the DDDDK sequence and cleaves the C-terminal peptide bond of the lysine residue. Due to its high reactivity at room temperature (temperature range of activity: 4–40 °C) and physiological pH (pH range of activity: 6–9) and mild reaction conditions (Collins-Racie et al. 1995), enterokinase is considered a powerful tool that widely applied in biotechnology and related sciences (Yuan and Hua 2002). The difficult process and high cost of extraction and separation of natural enterokinase and its probable contamination by other protease led to a great attempt to produce it on larger scale and lower finished cost in *E. coli*. A lot of research has been done to produce this enzyme in *E. coli* (Skala 2013; Chun et al. 2011) and *Pichia pastoris *(Smith 2013) in the form of IB (inclusion bodies) or soluble protein. Production of active enzymes in most of them was limited and needed several steps of purification or dialysis.

Thioredoxin (TRX) as a chaperon-like fusion tag helps the solubility and stability of the fusion protein (Berndt 2008). It can be followed by DDDDK enterokinase and cleaved from the target protein using enterokinase activity.

Different strains of *E. coli* have been developed for different purposes. Among them, Bl21 (DE3) with *Escherichia coli* T7 RNA polymerase-based protein production in combination with T7 promoter-based expression vector allows high-efficiency protein expression of any gene that is under the control of a T7 promoter. The lon protease deficiency and the lack of ompT, outer membrane protease, which can degrade recombinant proteins during purification serve as advantages of BL21 (Grodberg and Dunn 1988). NiCo21 (DE3) and SHuffle T7 strains have been also engineered to minimize *E. coli* protein contamination and recombinant protein folding containing multiple disulfide bonds in the cytoplasm, respectively (Robichon et al. 2011; Lobstein et al. 2012; Kim et al. 2021).

One of the complexities of enterokinase production is related to the multiple disulfide bonds and an unpaired thiol that directs the protein production process to the IB formation (Schumann and Ferreira 2004). One strategy to prevent the formation of inclusion bodies is to slow down the production of recombinant proteins at low temperatures because the ability of the protein to fold at temperatures above 30 °C is decreased. Sometimes, it is probably the easiest and the most useful method which can prevent inclusion body formation by facilitating correct folding. Reducing the temperature down to 20–25 °C during the induction phase favors the native state which is related to several factors, including a decrease in the driving force for protein self-association, a slower rate of protein synthesis, and changes in the folding kinetics of the polypeptide chain (Lobstein et al. 2012).

IPTG concentration is an important parameter that should be fine-tuned at the induction phase. In different studies, a wide range of IPTG concentrations has been used below 0.1 mM to more than 1 mM IPTG (Gasparian et al. 2003; Shoae et al. 2021). However, as IPTG is an expensive product, it is important to a use minimal inducer amount, necessary to obtain a higher transcription level by optimizing the process (Hansen et al. 1998). IPTG has two advantages over lactose: First, its uptake is not dependent on the Lac permease (it diffuses through the inner membrane), and second, it cannot be cleaved by β-galactosidase preventing turn-off of transcription (Schumann 2004).

Using response surface methodology (RSM) as a statistical method for analyzing a process lets the biotechnologists evaluate the impact of the main experimental variables and their effects on the recombinant protein production and explore the relationships between several explanatory and response variables to choose the best combinations of them and optimize the process conditions (Khuri et al. 2017).

In this research, we focus on increasing the production of the total amount of active recombinant human enterokinase light chain in the cell, then optimizing a simple method for its purification and formulation for long-term use.

## Materials and methods

Human enterokinase (Submitted accession number, OP810624, S1) light chain hEKL gene was designed with the help of codon optimization toward *Escherichia coli* codon preference and RBS designed with the RBS calculator (Salislab.NET/software). It was artificially synthesized with a thioredoxin (TRX) tag fusion partner at the N-terminal and a 5 His-tag peptide (5H) at the C-terminal by Biomatics Co. in the pET-15 expression vector. Host strains *E. coli* BL21(DE3), NiCo21 (C2529H), and SHuffle T7 Express (C3029H) BL21 (SHuffle T7 express) were purchased from BioLabs. The human enterokinase catalytic subunit was purchased from BioVision. Mini-preparation extraction kit was purchased from Bioscience. IDA resin Ni was purchased from Bioscience. PCR amplification kit was purchased from Amplicon. TRX-PTH peptide as a substrate for enterokinase was provided by Arya Tina Gene Co.

### Transformation and PCR analysis

The TRX-hEKL-pET-15 was used to transform the host strains *E. coli* BL21(DE3), NiCo21, and SHuffle T7 Express (BioLabs) by heat shock method and plated on the LB agar plate containing ampicillin in the final concentration 100 µg/ml. After overnight growth, the colonies were analyzed for the plasmid content. A single colony was inoculated in a 5 ml LB medium containing antibiotics ampicillin (100 µg/ml) and grown at 37^◦^ C overnight.

Plasmid DNA of transformed colonies was extracted by a mini-preparation extraction kit. PCR amplification was performed using a high-fidelity thermostable polymerase with a T7 promoter primer as a forward (5’ TAATACGACTCACTATAGGG 3’) and the T7 primer complementary to the 5’ end of the T7 terminator as a reverse primer (5’ GCTAGTTATTGCTCAGCGG 3’). After primary denaturation of 94 °C for 5 min, PCR cycles were as follows: denaturation at 94 °C for 1 min, annealing at 57 °C for 30 s, and extension at 72 °C for 1 min for 35 cycles and analyzed on a 1% TAE agarose gel.

### Experiment design

Different growth and induction conditions were studied using a statistical method, response surface methodology (RSM) in the form of a central composite design (CCD) for the prediction of the effective variables in the enterokinase expression. In the response level method, a model is defined for each response that examines the independent and interaction effects of variables on each response. The response was determined based on the bioactivity of the purified enzyme on its substrate. The concentration of purified enzyme was measured after purification by Bradford assay.

Seventeen treatments based on the central composite design, including three replications at the central point, were studied to optimize enterokinase production. The independent variables used in the first stage include the amount of OD (0.6, 1.2, and 1.8 AU) at 600 nm to determine the time of induction, IPTG concentration (0.2, 0.5, and 0.8 mM), and the type of bacterial strain. (SHuffleT7, BL21, NiCo21) (Table 1). In the design, OD of 1.2 at 600 nm, IPTG concentration of 0.5 mM, and SHuffleT7 strain type were at the central point. The maximum and minimum limit of each quantitative variable was chosen based on the reference data (Gasparian et al. 2003; Lobstein et al. 2012; Shoae et al. 2021; Kim et al. 2021).

**Table 1:** Experiment design by RSM

Level	Factor		
	OD	Host Cell	IPTG (mM)
1	0.6	BL 21	0.2
2	1.2	SHuffle	0.5
3	1.8	Nico	0.8

Response Surface Methodology (RSM) with 3 Factor			
Run	Factor 1: OD	Factor 2: Host Cell	Factor 3: IPTG
1	0.6	BL 21 (1)	0.2
2	0.6	BL 21 (1)	0.8
3	1.2	BL 21 (1)	0.5
4	1.8	BL 21 (1)	0.2
5	1.8	BL 21 (1)	0.8
6	0.6	SHuffle (2)	0.5
7	1.2	SHuffle (2)	0.2
8	1.2	SHuffle (2)	0.8
9	1.2	SHuffle (2)	0.5
10	1.2	SHuffle (2)	0.5
11	1.2	SHuffle (2)	0.5
12	1.8	SHuffle (2)	0.5
13	0.6	Nico (3)	0.2
14	0.6	Nico (3)	0.8
15	1.2	Nico (3)	0.5
16	1.8	Nico (3)	0.8
17	1.8	Nico (3)	0.2

### Growth and induction treatments

The transformed colony from each strain was grown at 37 °C overnight and inoculated into 100 ml fresh LB medium containing antibiotics ampicillin (100 ug/ml) using 2% of the final growth volume. All cultures grew at 37 °C up to the defined ODs and then induced by different concentrations of IPTG for 4 h in defined temperatures.

The cell pellet was harvested by centrifugation at 6000 rpm for 10 min, resuspended in 4 ml of lysis buffer containing Tris 50 mM PH:8, EDTA 1 mM, Na-deoxycolate 0.1 mg/ml, and then lysed by sonication. The supernatant and lysate pellet fractions were separated by centrifugation at 12,000 rpm for 20 min.

### SDS-PAGE and western blotting

Total soluble protein was quantified by Bradford assay and analyzed based on SDS-PAGE according to Laemmli 1970 with 6% stacking gel, and 15% separating gel and run at a constant current of 2.2 mA/cm. Samples were mixed with an equal volume of 2X sample loading buffer and treated by heating at 95 °C for 5 min in sample loading buffer before loading on gels. SDS–PAGE gels were stained with Coomassie blue R250. For western blot analysis, the separated polypeptides in acrylamide gel were transferred to nitrocellulose membrane and stained with the appropriate antibody mouse anti-his tag antibody, (Biolegend, 652,501) with a dilution ratio of 1:1000 and horseradish peroxidase-conjugated anti-mouse immunoglobulin G antibody (Sigma) with a dilution ratio of 1:2000. Finally, 3,3’- Diaminobenzidine (Sigma) was used to visualize the blots.

### Effect of induction temperature on enzyme production

After validating the suggested treatment of the RSM model to optimize the enzyme production in a separate experiment, the effect of induction temperature has been studied on active enzyme production at three levels of 18, 25, and 32 °C.

### Scale up of enzyme production

After the evaluation of the selected parameters, the process was scaled up in two steps. Firstly, an experiment was done in a one-liter culture volume in the flask and then a 10-L fermenter was used to check the production of the active enzyme in a higher amount. The parameters of fermentation were DO (density of oxygen) 30%, the temperature of 37 °C in the growth phase and 25 °C in the induction phase, IPTG 0.7 mM, and agitation 200–1000 rpm (New Brunswick).

One ml of glycerol stock was inoculated into a 50 ml LB medium containing 100 μg/mL ampicillin and incubated for 4–6 h at 37 °C under 150 r/min until OD_600_ reached to 0.6–0.8. Then it was transferred into a 450 L media flask overnight. Once the OD_600_ reached 3–4, it was transferred to a 10 L bioreactor containing 2.5 L of production medium consisting of 24 g/L of yeast extract, 12 g/L Tryptone, 4 ml glycerol, 12.5 g/L of di-potassium hydrogen orthophosphate, 23 g/L potassium di-hydrogen orthophosphate and 20 ml/L of trace salts (Holme et al. 1970). The pH was buffered to 6.0–7.0 by adding ammonia or phosphoric acid. When the OD_600_ reached about 80, the temperature was lowered to 30 °C and the expression of the fusion protein was initiated by the addition of 0.7 mmol/L IPTG. The incubation was maintained for 6 h. Cells were harvested by centrifugation at 8000 r/min for 20 min.

### Enzyme extraction, purification, and preservation

After cell lysis and centrifuge of each treatment, an autocatalytic reaction of the enzyme has been done in the supernatant for an overnight in the refrigerator.

The protein solution was loaded onto a Ni-IDA column equilibrated in the binding buffer containing phosphate buffer 50 mM pH 8, NaCl 450 mM, imidazole 2.5 mM. The protein solution was loaded onto the column after filtration in the ratio of about 5:1 (v/v) load to resin. The flow-through and elution fractions were analyzed by SDS-PAGE. After washing with three resin volumes of binding buffer, bound EK was eluted with three resin volumes of an elution buffer containing phosphate buffer 50 mM pH 8 NaCl 450 mM, imidazole 450 mM. The activity of the eluted enzyme was checked on the TRX linked by Asp–Asp–Asp– Asp–Lys to PTH substrate and analyzed by SDS-PAGE. About 2 mM CaCl_2_ and 40% glycerol were added to the active enzyme to preserve it at − 20 °C for long-term use.

### Enzyme bioassay

The kinetics of EK activity was measured by different amounts of substrate. Peptide substrate was dissolved to a concentration of 0.5 mM in 50 mM Tris–HCl, pH 8.0. One microliter of enzyme sample (4ug, 0.2 nM) was mixed with 95ul of substrate solution at different concentrations of 0.05, 0.1, 0.2, 0.4, 0.8 and 1 mM at 25 °C. Protein substrates were digested by EK in Tris 50 mM pH 8.0, NaCl 50 mM and CaCl_2_ 2 mM. The molar ratios of EK to target protein were varied. Digestion reactions proceeded for 15 h at room temperature (25 °C) and were monitored by SDS-PAGE.

Enzyme activity was measured by an increase in the peak area of PTH in the RP-HPLC system (Knauer, Germany) RP-1000 column, 8um, 250 × 4.6 mm at a flow rate of 1 ml/min with linear gradients of 10% to 95% acetonitrile containing 0.1% TFA as a counter ion.

Michaelis–Menten saturation curve was plotted based on the rate of formation of PTH to the concentration of a TRX-PTH. KM and vmax values were determined after linearization of the Michaelis–Menten saturation curve by Lineweaver–Burk plot.

## Results

**Fig. 1:**
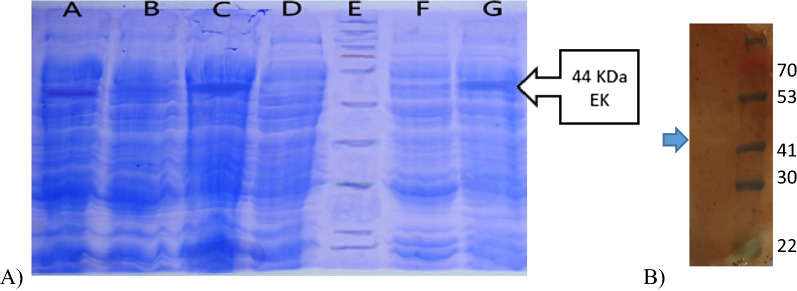
**A** Result of electrophoresis of total protein extracted from bacteria on SDS-PAGE gel. In this figure A = BL21, B = C^−^BL21, C = SHuffleT7, D = C^−^ SHuffleT7, E = Pr Ladder, F = C^−^NICO, G = and NiCo21. **B** Western blot of the impure protein sample

To compare the amount of the induced protein between the three strains, the total soluble protein was quantified by Bradford assay and analyzed based on SDS-PAGE immediately after cell harvest. Figure 1 SDS-PAGE results showed that the expression of target protein in fusion form (about 44 kDa) was induced in all three strains. The enterokinase polypeptide was visible on the western blot as a single band at approximately 44 kDa. To evaluate the effect of the fusion domain, the insoluble part of lysed cells was dissolved in urea 8 M and was quantified by Bradford assay and the ratio of insoluble to soluble was below 5%.

**Fig. 2:**
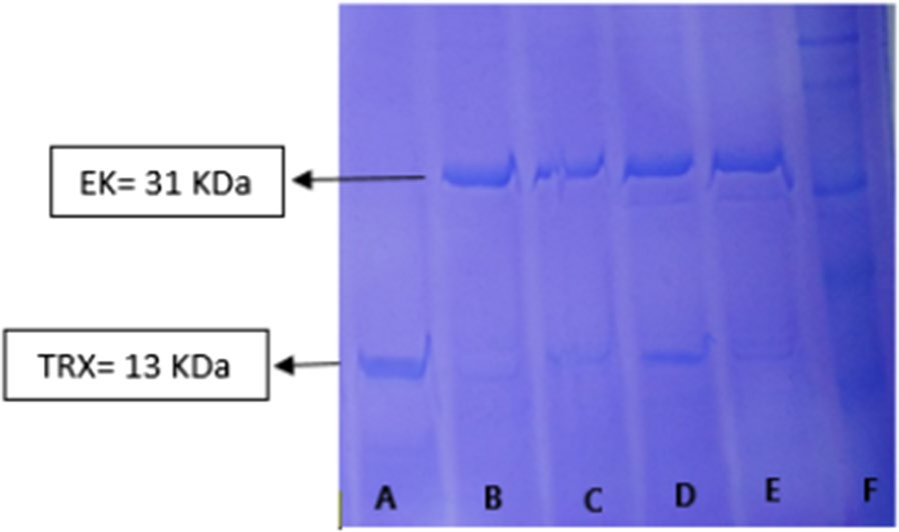
Purification of separated TRX from the active enzyme by IDA affinity chromatography after the autocatalytic reaction. A = bind fraction, B, C = elution peak fractions, D,E = different fractions of elution, F = Pre-stained protein ladder

After an overnight in the refrigerator, the autocatalytic process cleaved the TRX tag with the enterokinase recognition site by the unpurified hEK and yield the mature enzyme (Fig. 2).

**Fig. 3:**
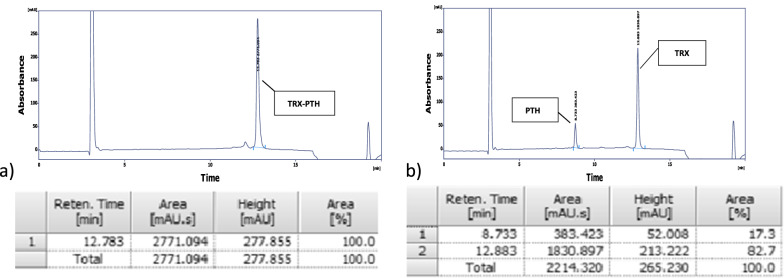
HPLC analysis of the enzyme reaction on TRX-PTH substrate, **a** undigested substrate, **b** digested substrate

Determining the appropriate range of independent variables of enterokinase production based on the reference data (Niu et al. 2014) (including OD of induction among 0.6, 1.2, and 1.8, IPTG concentration among 0.2, 0.5, and 0.8 mM, and type of bacterial strain among Bl21, Nico and SHuffle), the response surface method (RSM) were used in the form of a central composite design to predict independent variables on enterokinase production (Table 1). The response level was measured based on enzyme activity on TRX-PTH substrate and the peak area of PTH in HPLC the calculated concentration of the PTH peak based on calibration data was 0.14 mg/ml (Fig. 3).

The activity of hEKL was measured based on the peak area of PTH peptide in HPLC during digestion reaction on TRX-PTH peptide as a substrate with the retention time of 12.7 min, PTH, and thioredoxin as products of the reaction with the retention time of 8.7 and 12.9 min respectively.

In the response level method, a quadratic model significantly defined the independent and interaction effects of variables on each response (Table 2).

**Table 2:** Investigation of the independent and interaction effect of variables on each response

Source	Sum of squares	DF	Mean square	F value	Prob > F
Mean	7.696E + 010	1	7.696E + 010		
Linear	2.304E + 009	3	7.680E + 008	0.26	0.8543
2FI	1.638E + 009	3	5.460E + 008	0.15	0.9290
*Quadratic*	*2.486E + 010*	*3*	*8.287E + 009*	*4.76*	*0.0410*
Cubic	9.917E + 009	4	2.479E + 009	3.28	0.1785
Residual	2.270E + 009	3	7.567E + 008		
Total	1.179E + 011	17	6.938E + 009		

**Fig. 4:**
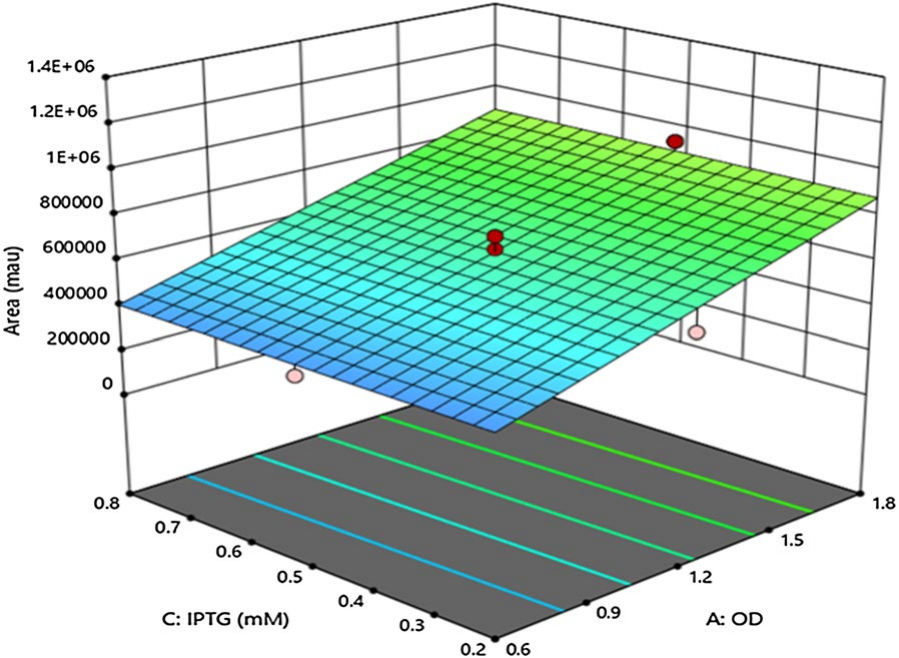
Interaction effect of A: optical density (OD), C: IPTG concentration, Shuffle T7 strain has been selected as actual factor in 3D surface plot

Based on the quadratic model, the three dimensional curve has been plotted for the variables of IPTG concentration and OD. Shuffle T7 strain has been selected as actual factor. In this plot, the effects of all the factors at a particular point in the design space were compared. The model makes a relationship between the inputs and the outputs of the system which objective is the system description (Fig. 4).

The maximum yield of the active enzyme was predicted to be obtained from the Shuffle T7 strain at OD of 1.8 with 0.71 mM IPTG as an inducer.

The result of the repeated experiment with these parameters led to the production of the active enzyme with the product peak area of 1503 mAU in HPLC (95%). The confirmed result from the model predictions was used in the next experiment as constant parameters. Based on the optimal time of induction based on OD, the optimal concentration of IPTG, and Shuffle T7 strain as the best host discovered in the prior experiment, the second experiment was done on induction temperature at three levels of 18, 25, or 32 °C.

**Fig. 5:**
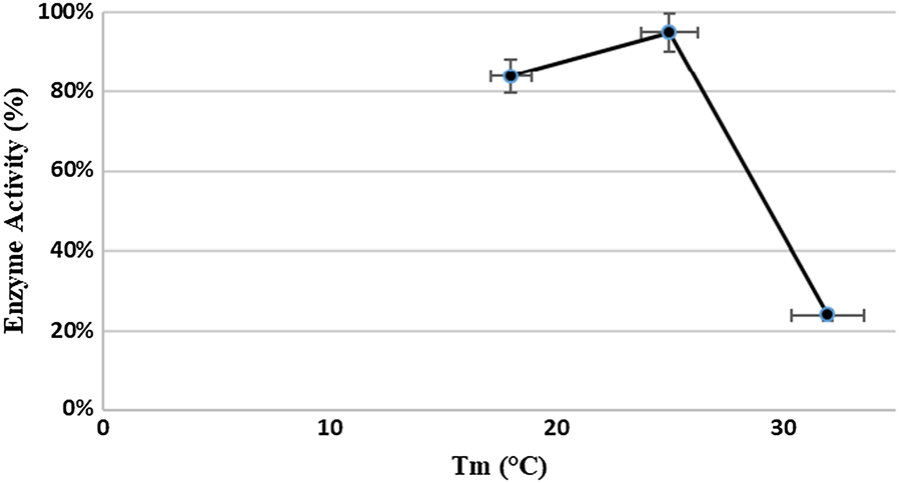
Effect of induction temperature on enzyme production based on PTH peak area during enzyme bioassay. The temperature treatments had three replicates

According to Fig. 5, the temperature of the induction phase had a significant effect on the bioactivity of the enzyme. The maximum activity of the enzyme and the highest PTH area were related to the enzyme that was expressed at 25 °C and the lowest one was related to the 18 °C treatment.

After confirmation of the production of active enzyme based on the defined treatments in a larger volume of 1 L in the scale-up process, all selected parameters were applied to a 10-L fermenter. The amount of 80 mg pure active hEKL per 1 L culture was obtained with a specific activity of 6.25 × 10^2^ U/mg. One unit (AU) was arbitrarily defined as 0.045 ug of enterokinase which cleaves 100 ug of test substrate to 95% completion in 16 h or less at 25 °C (data not shown).

### Enzyme bioassay

The activity of hEKL was measured based on the peak area of PTH peptide in HPLC during digestion reaction on TRX-PTH peptide as a substrate. The activity of hEKL was compared to a commercial enzyme of BioVision (Catalog # K760-100) with the approximately same result.

Based on the BioVision datasheet, one unit of the enzyme is about 83 µg of the enzyme needed for cleavage of 1 µmol of substrate. For TRX-PTH peptide with the molecular weight of 18 KDa, 250 ng of BioVision enzyme (about 0.5 µl) were used for 100 µg substrate in 16 h or less at 25 °C.

**Fig. 6:**
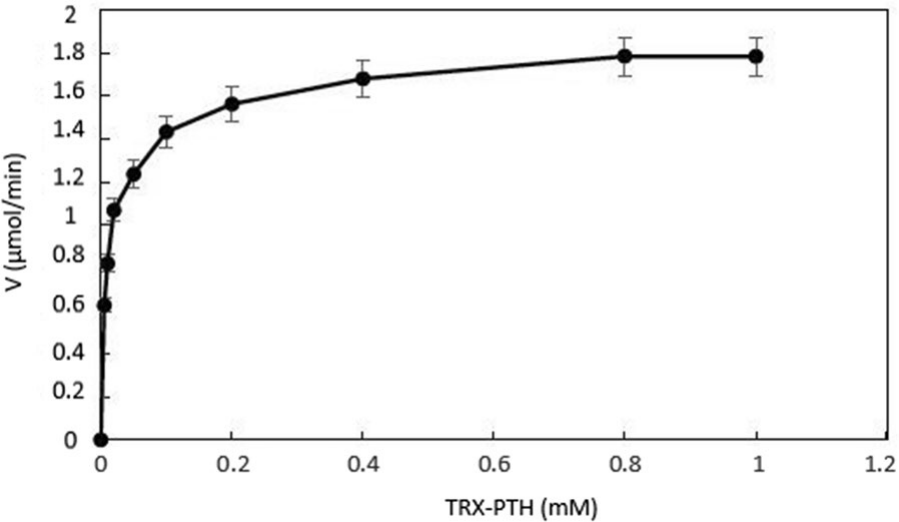
Michaelis–Menten saturation curve for the enzyme reaction

**Fig. 7:**
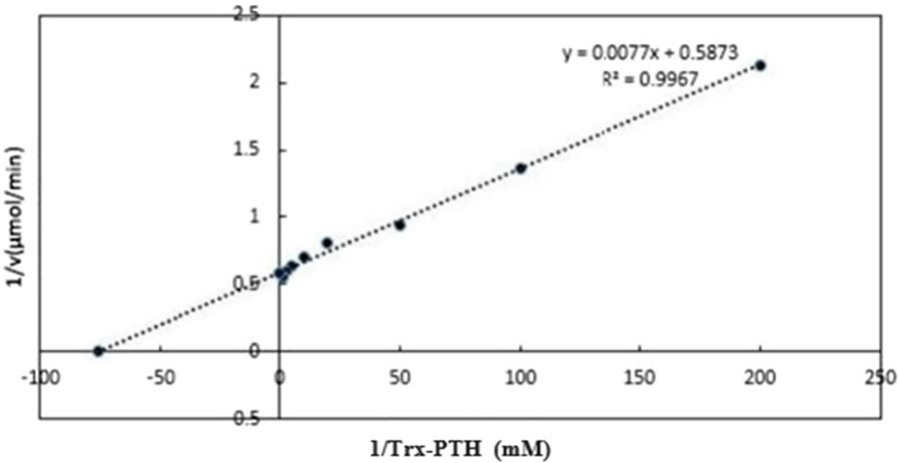
Linearized Michaelis–Menten saturation curve by Lineweaver–Burk plot

Michaelis–Menten saturation curve for the enzyme reaction showed the relation between the substrate concentration (TRX-PTH) and reaction rate (Fig. 6). Linearization of the equation was done based on Lineweaver–Burk plot. The calculated Km based on the linearized plot was 0.013 mM and Vmax was 1.7 (Fig. 7).

## Discussion

Production of recombinant entrokinase has been studied in the form of soluble or insoluble protein for many years but there are still challenges to produce it in an active form. Cytoplasmic, periplasmic, or inclusion body production of this enzyme in *E. coli* (Chun et al. 2011; Huang et al. 2007; Ayat et al. 2022; Nanjundaiah et al. 2021; Phuong et al. 2021) or secretion production in *Pichia pastoris* (Melicherova et al. 2017) has been reported by many researchers. In this study, we could optimize the cytoplasmic production of active enzyme simply and efficiently in a large volume considering that in our previous attempt for the enzyme production in inclusion body form, the results of the correctly folded enzyme were not acceptable (data not shown).

Expression of the recombinant enterokinase catalytic subunit with TRX tag and DDDDK recognition sequence in N-terminal in *Escherichia coli* causes most products to exist in soluble form and an in vivo autocatalytic cleavage of the TRX–EK catalytic domain leads to intact, biologically active EK catalytic subunit. The autocatalytic ability of enterokinase to make it free from the fusion tag and as proof of its bioactivity has been studied in many types of research (Collins-Racie et al. 1995; Yuan and Hua 2002; Gasparian et al. 2003; Chun et al. 2011; Kwon et al. 2021).

The primary results showed that the Shuffle strain had the most active enzyme compared to two other strains. Considering the nine cysteines in the enterokinase light chain peptide and four disulfide bonds in its structure, it is a problematic peptide to express in an active form in the cytoplasm. SHuffle strain is a special host cell for its diminished cytoplasmic reductive pathways and its cytoplasmically over-expressed disulfide bond isomerase (DsbC) that allow for the formation of disulfide bonds in the cytoplasm. Therefore, it was an ideal host cell for such a complicated peptide to express in the cytoplasm (Lobstein et al. 2012). Simultaneously placing a thioredoxin tag in front of the peptide sequence in the designed cassette as an important solubilizing and stabilizing agent, was completely effective for the solubility and correct folding of the protein.

Thioredoxin as a redox-active helper protein is one of the most successful classes of helper proteins. Lobstein et al. (2012) reported that redox-active helper proteins had the biggest effect on increasing the capacity of the cells to produce correctly folded disulfide-bonded proteins.

The advantage of cytoplasmic expression was observed in the case of a truncated form of the human tissue plasminogen activator vtPA polypeptides with very complex patterns of nine disulfide bonds, which had a 15-fold increase in activity by co-expression of TrxA in the cytoplasm (Bessette et al. 1999). In another study, the activity of α1, 3 galactosidase from *Xanthomonas manihotis* with a single disulfide bond was increased sevenfold in the cytoplasm instead of the periplasm (Lobstein et al. 2012).

Investigation of the effect of different concentrations of IPTG inducing at various growth phases revealed that increasing OD to the late logarithmic phase and decreasing IPTG concentration led to more active enzyme production. It implies that the decrease of the total amount of the recombinant protein expression in a single cell can be helpful for the folding system not to be saturated and prevent the toxicity of the over-expressed poorly folded proteins with a complex structure while the number of the cells by higher OD compensates for the amount of the protein product. However, Shoae et al. (2021) reported the optimized condition of 1.05 mM IPTG at OD600 of 0.6 for recombinant bovine light chain enterokinase in *E. coli* (Shoae et al. 2021).

Considering the interaction effect of the OD of induction with IPTG concentration, inducing at various growth phases by different amounts of IPTG affects the expression of the soluble proteins with multiple disulfide bonds (Lobstein et al. 2012).

The effect of temperature on protein folding is one of the most important factors to be optimized during the production of proteins (Schein et al. 1991). We investigated the role of temperature on protein expression in SHuffle cells during induction by shifting the growth temperature from 37 °C to 18 °C, 25 °C, or 32 °C.

The optimal temperature varied among the proteins as well as the host cells. In the case of SHuffle cells that are under oxidative stress, the resulting detrimental effects may be compounded by high metabolic activity during the induction period at high temperatures such as 37 °C. This is supported by the observation of over-expression of poorly folding proteins such as vtPA at 37 °C in SHuffle cells (Lobstein et al. 2012).

According to Ren et al. (2016), if the expression of the protein of interest, is a metabolic burden because of multiple disulfide bonds, lowering the temperature increased the expression of the correctly folded protein. So it is recommended to grow and express that protein in SHuffle cells at 30ºC or below. At low temperatures, the growth rate of the bacterium decreases, although it slows down the rate of protein synthesis and folding kinetics, it decreases the hydrophobic interactions that are involved in protein self-aggregation and led to an increase of recombinant proteins in the soluble form and a decrease in the recombinant protein in the form of inclusion bodies (Schumann et al. 2004).

In conclusion, the gene expression of enterokinase was studied in SHuffle T7, BL21, and NiCo21 strains of *Escherichia coli*. The highest expression of active enterokinase happened in the SHuffle T7 strain in OD of induction equal to 1.2 at 600 nm and IPTG concentration of 0.71 mM in LB culture medium at 25° C induction temperature. Using the TRX fusion tag in combination with SHuffle T7 strain as a host cell and optimizing the growth condition simultaneously effectively led to the soluble, correctly folded, and bioactive enterokinase. We described the importance of choosing a suitable host cell beside the induction conditions for production of active enzyme in *E. coli* with all benefits of speed, cost and yield.

## Data Availability

The datasets generated during and/or analyzed during the current study are not publicly available because the found for the production of recombinant hEK was prepared by Arya Tina Gene company and some data should be patent for it. But are available from the corresponding author on reasonable request.

## References

[CR1] Ayat H, Darvishi O, Moazeni E, MomeniBidezard A (2022). Comparison of periplasmic and cytoplasmic expression of bovine enterokinase light chain in *E. coli*. The Protein.

[CR2] Berndt C, Lillig CH, Holmgren A (2008). Thioredoxins and glutaredoxins as facilitators of protein folding. BBA.

[CR3] Bessette PH, Åslund F, Beckwith J, Georgiou G (1999). Efficient folding of proteins with multiple disulfide bonds in the *Escherichia coli* cytoplasm. PNAS.

[CR4] Chun H, Joo K, Lee J, Shin H-C (2011). Design and efficient production of bovine enterokinase light chain with higher specificity in *E. coli*. Biotechnol Lett.

[CR5] Collins-racie LA, McColgan J, Grant JM, DiBlasio-Smith EA, McCoy JM (1995). Production of recombinant bovine enterokinase catalytyc subunit in *E.coli* using the novel secratory fusion partner DsbA. Nature Biotech.

[CR6] Gasparian ME, Ostapchenko VG, Schulga AA, Dolgikh DA, Kirpichnikov MP (2003). Expression, purification, and characterization of human enteropeptidase catalytic subunit in *Escherichia coli*. Protein Expr Purif.

[CR7] Grodberg J, Dunn JJ (1988). OmpT encodes the *Escherichia coli* outer membrane protease that cleaves T7 RNA polymerase during purification. J Bacteriol.

[CR8] Hansen LH, Knudsen S, Sorensen SJ (1998). The effect of a lacY gene on the indection of IPTG inducable promoters in *Escherichia coli* and *Pseudomonas fluorescens*. Curr Microbiol.

[CR9] Hayat SM, Farahani N, Golichenari B, Sahebkar A (2018). Recombinant protein expression in *Escherichia coli* (*E. coli*): what we need to know. CPD.

[CR10] Holme T, Arvidson S, Lindholm B, Pavlu B (1970). Enzymes: laboratory-scale production. Process Biochem.

[CR11] Huang L, Ruan H, Gu W, Xu Z, Cen P, Fan L (2007). Functional expression and purification of bovine enterokinase light chain in recombinant *Escherichia coli*. Pre Biochem Biotech.

[CR12] Kaur J, Kumar A, Kaur J (2018). Strategies for optimization of heterologous protein expression in *E. coli*: Roadblocks and reinforcements. IJBM.

[CR13] Khuri AI (2017). Response surface methodology and its applications in agricultural and food sciences. BBIJ.

[CR14] Ki M, Pack SP (2020). Fusion tags to enhance heterologous protein expression. Appl Microb Biotech.

[CR15] Kim Y, Lee H, Park S, Kim Y, Ahn J (2021). Expression and purification of soluble and active human enterokinase light chain in *Escherichia coli*. Biotech Rep.

[CR16] Kwon J, Choa H, Kim S, RyubJoong Y, Lee J (2021). A combination strategy of solubility enhancers for effective production of soluble and bioactive human enterokinase links open overlay. J Biotech.

[CR17] Laemmli UK (1970). Cleavage of structural proteins during the assembly of the head of bacteriophage T4. Nature.

[CR18] Liew OW, Chong JPC, Yandle TG, Brennan SO (2005). Preparation of recombinant thioredoxin fused N-terminal proCNP: analysis of enterokinase cleavage products reveals new enterokinase cleavage sites. Protein Expr Purif.

[CR19] Lobstein J, Emrich CA, Jeans C, Faulkner M, Riggs P, Berkmen M (2012). SHuffle, a novel *Escherichia coli* protein expression strain capable of correctly folding disulfide bonded proteins in its cytoplasm. MCF.

[CR20] Melicherová K, Krahulec J, Šafránek M, Lišková V, Hopková D, Széliová D, Turňa J (2017). Optimization of the fermentation and downstream processes for human enterokinase production in *Pichia pastoris*. Appl Microb & Biotech.

[CR21] Nanjundaiah SN, Ma J, Sukumaran S, Sambasivam G (2021). Cloning, Expression, and Purification Strategies for Enhanced Production of Enterokinase using TrpE fusion tag in Bench Scale Bioreactor. J Appl Biotech Rep.

[CR22] Niu LX, Li JY, Ji XX, Yang BS (2014). Efficient expression and purification of recombinant human Enteropeptidase light chain in *Escherichia coli*. BABT.

[CR23] Phuong K, Huyen DT, Hong LTT (2021). Purification and determination of the biological activity of a recombinant enterokinase. Vietnam J Biotechnol.

[CR24] Ren G, Ke N, Berken M (2016). Use of the SHuffle Strains in Production of Proteins. Curr Proto Protein Sci.

[CR25] Robichon C, Liu J, Causey T, Benner JS, Samuelson JC (2001). Engineering *Escherichia coli* BL21(DE3) derivative strains to minimize E coli protein contamination after purification by immobilized metal affinity chromatography. Appl Environ Microb.

[CR26] Schein CH (1991). Optimizing protein folding to the native state in bacteria. Curr Opin Biotechnol.

[CR27] Schumann W, Ferreira CS (2004). Production of recombinant proteins in *Escherichia coli*. Genet Mol Biol.

[CR28] Shoae M, Safarpour H, Khorashadizadeh M (2021). Recombinant production of bovine enteropeptidase light chain in SHuffle® T7 express and optimization of induction parameters. Protein J.

[CR29] Skala W, Goettig P, Brandstetter H (2013). Do-it-yourself histidine-tagged bovine enterokinase: A handy member of the protein engineer’s toolbox. J Biotechnol.

[CR30] Smith ET, Jhonson DA (2013). Human enteropeptidase light chain: Bioengineering of recombinants and kinetic investigations of structure and function. Protein Sci.

[CR31] Yuan LD, Hua ZC (2002). Expression, purification, and characterization of a biologically active bovine enterokinase catalytic subunit in *Escherichia coli*. Protein Expr Purif.

